# Supplementary material for: Influence of running shoes on muscle activity
Source: PLoS One. 2020 Oct 7;15(10):e0239852. doi: 10.1371/journal.pone.0239852 (PMC7540877; doi:10.1371/journal.pone.0239852)
Supplement: Attachment — Submitted filename: Response to Reviewers.docx [file pone.0239852.s002.docx]

**Academic Editor Comments**

1. Please ensure that your manuscript meets PLOS ONE's style requirements, including those for file naming.

The manuscript and all submitted documents have been revised to adhere to the journals style requirements and naming conventions.

2. In your revised cover letter, please address the following prompts:
   a. If there are ethical or legal restrictions on sharing a de-identified data set, please explain them in detail (e.g., data contain potentially identifying or sensitive patient information) and who has imposed them (e.g., an ethics committee). Please also provide contact information for a data access committee, ethics committee, or other institutional body to which data requests may be sent.
   b. If there are no restrictions, please upload the minimal anonymized data set necessary to replicate your study findings as either Supporting Information files or to a stable, public repository and provide us with the relevant URLs, DOIs, or accession numbers. Please see http://www.bmj.com/content/340/bmj.c181.long for guidelines on how to de-identify and prepare clinical data for publication. For a list of acceptable repositories, please see <http://journals.plos.org/plosone/s/data-availability#loc-recommended-repositories>.

The minimal anonymized data set necessary to replicate the findings presented in this study have been uploaded as a supporting information file.

3. We note that you received funding from a commercial source: Mizuno and Biomechanigg Sport. Please provide an amended Competing Interests Statement that explicitly states this commercial funder, along with any other relevant declarations relating to employment, consultancy, patents, products in development, marketed products, etc. Within this Competing Interests Statement, please confirm that this does not alter your adherence to all PLOS ONE policies on sharing data and materials by including the following statement: "This does not alter our adherence to PLOS ONE policies on sharing data and materials.” (as detailed online in our guide for authors <http://journals.plos.org/plosone/s/competing-interests>). If there are restrictions on sharing of data and/or materials, please state these. Please note that we cannot proceed with consideration of your article until this information has been declared. Please include your amended Competing Interests Statement within your cover letter. We will change the online submission form on your behalf.

Please know it is PLOS ONE policy for corresponding authors to declare, on behalf of all authors, all potential competing interests for the purposes of transparency. PLOS defines a competing interest as anything that interferes with, or could reasonably be perceived as interfering with, the full and objective presentation, peer review, editorial decision-making, or publication of research or non-research articles submitted to one of the journals. Competing interests can be financial or non-financial, professional, or personal. Competing interests can arise in relationship to an organization or another person. Please follow this link to our website for more details on competing interests: <http://journals.plos.org/plosone/s/competing-interests>

A competing interest statement has been amended to the cover letter.

**Reviewer 1 Comments**

**General**

This study investigated the effects of footwear on kinematics and muscle activity in runners. This is an important topic, as rates of running injuries have not declined in past decades, despite a large body of biomechanics and footwear research. The authors investigated an alternative paradigm which could change how shoe design is approached. The manuscript is well-written, and overall the study design is strong. I do have questions regarding the author’s choice of definition of “maintained” vs. “changed” kinematics, which may impact the interpretation and meaning of the results, which I have included in my comments below.

**Specific**

ABSTRACT

Line 25: I would consider this a repeated measures design, since within subject comparisons are analysed.

The authors agree with the reviewer opinion that this study should be considered a repeated measures design. The strong revisions applied to the manuscript, however, resulted in an entirely updated abstract which does not touch on the type of design the study incorporated.

INTRODUCTION

Lines 56-57: This statement makes in unclear how the preferred movement path might influence injury or shoe design. If runners naturally use their preferred movement path, why do they still experience injury? And if they maintain this path in different footwear conditions, does that mean shoe design is not important? If you could, provide a rational for how you see this informing shoe design/reducing injury risk in the future.

The authors thank the reviewer for this comment. It touches on one of the main difficulties of the PMP paradigm. According to Nigg et al. (2017), a runners PMP is not necessarily constant, but rather dependent on many factors (e.g., injury, training status, footwear, etc.). It is speculated that the locomotor system of a given runner aims to maintain a current PMP as it has potential to prevent/reduce injuries. Footwear, however, is currently not designed to help a locomotor system to maintain a beneficial PMP and can as result counteract the system’s attempt to maintain a beneficial PMP. As such, current footwear design might in fact be harmful. It is of high importance, therefore, to identify/develop footwear designs that support a given PMP and help the locomotor system in its attempts to reduce injury and/or energy demands.

The manuscript has been revised to clarify this interaction between PMP, footwear, and injury in lines 62-64.

Lines 60-61: Please specific that is was changes in footwear that lead to these changes in kinematics.

The revised manuscript addresses this comment in line 70, stating that changes in footwear lead to changes in kinematics.

Lines 63-65: What is the rationale for 3 degrees? Studies associating kinematics with injury often finding differences between injured and healthy groups at discrete time points of just 3-4 degrees.

The original work by Nigg et al. (2017) states three thresholds: 2°, 3°, and 5°. The argument for the 2° threshold is that differences in joint movement below this threshold fall below the degree of reliability for skin marker-based 3D motion capture. The above work by Nigg and colleagues also highlights that the results for the 3° and 5° thresholds are essentially identical, independent of the threshold chosen. While summarizing the results of previous works, the authors chose to report the more conservative threshold of 3° as many studies comparing different running shoes often report kinematic differences between 1° and 3°. The somewhat arbitrary choice of a 3º threshold is scrutinized within the discussion section of the revised manuscript.

METHODS

I realize the methods have been described in a previous publication. However, it would be relevant to include here the pace participants ran at, for how long, and how many steps were included in analysis.

The revised manuscript states that participants performed 10 running trials per condition at 3.3 m/s (± 15 %) in line 131. For further clarification it was added that one running trial was approx. 10 steps. The steps included in the analysis varied from participant to participant. An appropriate statement was added in line 161.

Line 94: Change “wider” to “wide”.

Changes have been adapted in line 122 in the revised manuscript.

Line 128: Were specific criteria for detecting outliers used? How many trials were removed?

Across participants, the number of removed trials varied. However, for all participants not more than five trials were removed. A respective statement can be found within line 161 in the revised manuscript.

Line 134: Could a statistical method, such as used to determine changes in EMG activation, be used to decide whether kinematics changed?

This comment refers to the support vector machine method applied in the initial manuscript. The revised manuscript, however, applies an entirely different methodology and the authors feel that the comment is therefore not applicable anymore. From a theoretical point of view, however, the answer to the question posed by the reviewer is yes.

Also, using the current method, what is the clinical relevance of a mean absolute difference of 2° across the stance phase? What was the maximum difference at any given timepoint during stance? A larger difference, let’s say at midstance, of 4-5 degrees, which may be clinically significant, when averaged over stance could possibly still result in a mean absolute difference less than 2. Showing kinematic trajectories of an exemplary subject in the different footwear conditions might help demonstrate similarity in movement patterns.

The revised manuscript provides exemplary kinematic trajectories for all analysed trials of a representative participant (Fig 2 and 3). To address the reviewer’s comment, a mean absolute difference of 2º is potentially not relevant form a clinical perspective. It is, however, the reliability threshold of marker-based motion capture systems and the smallest quantifiable difference possible. The revised manuscript chose a threshold of 3º, as a compromise between reliable and accurate measurements, and clinical relevance (approx. 4º).

Line 144: Summing the corresponding result across what?

Summing across the individual wavelets. The paragraph describing the individual analysis steps has been extensively updated. In the revised manuscript lines 182-189 describe the procedure the comment referred to.

DISCUSSION

Line 203: What about hip kinematics? Hip kinematics were included in the methods.

While reflective markers were placed at the hip, kinematic movement trajectories have been calculated for the ankle and knee joint only. The authors thank the reviewer for point towards this confusion. Care has been taken through the entirety of the revised manuscript to avoid said confusion.

Lines 204-205: I assume most if not all runners in this study were habitually shod. As a novel task, it would be expected that barefoot running would elicit increases in muscle activity. This should be addressed. What do the authors speculate would happen if runners were given an extended adaptation period?

Given that a preferred movement path is not constant but rather dependent on various factors, the authors would speculate that runners exposed to an extended adaptation period would eventually find their optimal preferred movement path in barefoot running. With respect to the revised manuscript, the authors would like to point out that the results and discussion section have been entirely rewritten. The revised version includes muscle specific changes in activation, addressing the reviews comment.

**Reviewer 2 Comments**

**General**

The purpose of this study was to compare kinematics and EMG activity during running between 4 conditions, 3 shod and 1 barefoot with respect to the preferred movement paradigm. While basic data collection procedures were sound, I have major concerns over the study justification, data analysis and statistical methods, the presentation of results, and the proposed implications of these results. Major revisions to this article would be needed for publication consideration. Please see the attached file for specific comments.

The authors thank the reviewer for the assessment of the manuscript and the suggestions for revisions. As suggested by the reviewer, major revisions have been performed. Methodologies were adapted, results novel results were produced/presented, and the discussion has been entirely rewritten. The revised document addresses the individual comments of the reviewer.

**Specific**

The purpose of this study was to compare kinematics and EMG activity during running between 4 conditions, 3 shod and 1 barefoot with respect to the preferred movement paradigm. While basic data collection procedures were sound, I have major concerns over the study justification, data analysis and statistical methods, the presentation of results, and the proposed implications of these results. Major revisions to this article would be needed for publication consideration.

**Major Comments**

1. While I agree that running shoe development over the past 50 years has failed to make significant advances in decreasing running injury frequency, I disagree that looking at ground reaction forces and eversion kinematics is now somehow frivolous, as there are numerous studies that have linked factors in these categories to injury, including many prospective studies. I also agree that runners likely have a preferred movement path (i.e. their barefoot running mechanics) that shoes influence, and that most modern running shoes are over-engineered and may push runners away from this path. From this, the question I believe you would want to ask is: do runners change their preferred movement path (i.e. change kinematics and/or EMG) between barefoot and shod conditions? This question would directly address whether shoes alter a runner’s PMP. However, this is not exactly how you structured your study, and your justification and discussion of your results does not follow this rationale. Either, I think you need to restructure your study and only focus on barefoot vs shod, or you need to better explain the rationale for your current design, as I fail to see what the shod vs. shod comparison (2 of the shoes being very similar in design) shows you about the preferred movement path hypothesis.

The authors thank the reviewer for their assessment. The revised manuscript states that older paradigms (i.e., impact force and pronation) were derived from an incomplete functional understanding of running kinematics. This does not suggest that ground reaction forces and eversion kinematics should no longer be investigated. It merely proposes that the way research has tried to interpret these variables and link them to running injuries/frequencies might not have been appropriate. In fact, the authors believe that one should continue to investigate ground reaction forces and eversion kinematics but approach their interpretation from a functional biomechanical perspective. With the aim to clarify this confusion, the paragraph has been changed (line 52-54).

The authors believe that the alternative question posed by the reviewer (if runners change their PMP between barefoot and shod) is certainly a valid one. Following the definition of the PMP paradigm, however, this question has theoretically been answered and was therefore not the focus of the present work. As stated in Nigg et al. (2017), the paradigm proposes that a PMP is not exclusively an individual’s barefoot running mechanics. Rather, a PMP is dependent on factors such as training status, fatigue, injury, and footwear. In other words, on a given day a runner has a specific PMP during barefoot running. This PMP, however, may change when compared to a different day. Further, when introducing footwear, the PMP of the given individual will, according to the definition of the paradigm, change as the constraints have changed. Thus, the question one would have to ask is how to identify/quantify a given PMP. As it has been proposed that muscle activity might provide insights to this question, the present manuscript aimed to investigate said proposal by comparing muscle activity in various (similar) footwear conditions that would not change an individual’s PMP and barefoot which would change and individuals PMP. The authors do agree with the reviewer that this objective has not been stated to a clear and satisfactory degree. The manuscript has been adjusted accordingly.

Along the same lines, I think your study may be stronger if you de-emphasize the preferred movement hypothesis in your rationale, and simply compare the 4 conditions using traditional statistical techniques (ANOVAs). This would be a much cleaner and digestible study that the analysis that was actually performed. You could still discuss the implications of these results in light of the preferred movement paradigm, and the results would be more generalizable to the overall body of running injury literature. Right now, your study essentially shows that runners change kinematics and EMG between shod and barefoot, but not between shod conditions, which I don’t see as a very compelling or linear argument for the PMP paradigm.

The authors acknowledge that the analysis procedures have been presented in a difficult way. The revised manuscript includes a novel approach to the statistical analysis. The purpose of the manuscript, however, is to specifically address the paradigm of the preferred movement path. While the initial submission did not work well towards this goal, the authors feel that the revised manuscript justifies the updated approach.

2. I am confused by the shoes selected for this study. The Mizuno Be is not a running shoe, it is a shoe designed for runners, but not for running (confusing, I know…). Why use it here? Furthermore, despite your explanation, I do not understand the difference between this shoe and the Wave Universe. They are both minimal in design. Why not use a maximally cushioned shoe as your 3rd shoe condition? More explanation is needed here as to what the Mizuno Be added to your study in terms of the potential study implications.

The shoes were selected with the intention to have two similar (but not identical) models and one rather different model. The Mizuno Be and the Mizuno Wave Universe are similar in their minimalist design, yet somewhat different. Considering the paradigm of the preferred movement path, a change between these two models represents minimally changed boundary conditions / constrains. As such, the paradigm suggests that a change between these two models would result in minimal adaptations because in a system’s preferred movement path would remain the same. The Mizuno Rider and the Barefoot condition on the other hand represent more substantial changes in boundary conditions (Barefoot more so than the Mizuno Rider) and it was of interest to investigate how this affects a runner’s preferred movement path. From that perspective, this study included two similar running conditions (Be and Universe), one slightly different (Rider), and one entirely different (barefoot). As the focus of this study was placed on changes in EMG activity, it was of interest to have this variability in running conditions.

3. Why did you look at EMG power spectrum instead of simply looking at EMG amplitude and/or timing? Looking at the average EMG amplitude normalized by MVC would allow you to investigate whether runners increased/decreased/maintained the average muscle activity between shoes. In addition, looking at EMG timing (onset relative to footstrike, duration, offset relative to toe-off) would be very appropriate here, as this would tell you whether runners changed their neuromuscular recruitment strategies between shoes. Either of these techniques would make your results much more digestible and easier to comprehend. The EMG analysis technique you used are convoluted and likely only understood by a very small fraction of readers. I think you overcomplicated what should have been simple. If you decide to keep your current analysis, you need to justify the rationale for this technique and explain what information this gives you that simpler techniques would not.

The authors agree with the reviewer that the methods presented in the initial submission were convoluted. To address this problem, the revised version highlights changes in the area under the curve of EMG intensity signals. With this approach the manuscript provides a tangible and clear method to compare changes in EMG activity. The authors thank the reviewer for the suggestions that inspired the restructuring of the manuscript.

4. Your results section is insufficient. Is not acceptable to simply insert a table with the number of runners who maintained/changed their kinematics or EMG. You need to report the means/SDs for your data, along with effect sizes if appropriate. I cannot accept this paper for publication without this change, as your data lacks both tangibility and transparency.

The entire results section has been rewritten. Further, the statistical procedures used within this manuscript have been revised.

**Minor Comments by Section**

Abstract

1. Objectives- Writing is unscientific – please revise without /’s

The suggested changes have been adapted.

2. Results - I am confused by the results, shouldn’t the percents be an exact number and not a range? It will probably make more sense as I read the full results section, but I should not need to read the full results section to understand the abstract. Please revise.

The entire results section has been rewritten and extended.

Introduction

1. The vast majority of references on PMP are from your research group, which creates concern of scientific bias. This is a borderline major comment for me, as the Nigg research group and its disciples are predominately (exclusively?) the ones pushing the PMP paradigm. Are there other research groups that have done similar work on PMP that you can cite?

The authors agree with the reviewer’s assessment that the main body of literature published on the topic of the preferred movement path paradigm is in fact by the Nigg research group. While the authors acknowledge that this can pose a significant research bias, the authors are unsure how to address this issue as scientific literature on the preferred movement path is scarce. To the knowledge of the authors, in 2019 a single study has been published outside of the Nigg research group. Trudeau et al. (2019) addressed the paradigm of the preferred movement path to inspire the idea of the habitual motion path. This study, however, was not published when the present manuscript was first prepared. It has been added as part of the revision process. Although studies on the preferred movement path paradigm a sparse, many authors have provided comments on the topic. For instance, Vanwanseele and colleagues (2018) or Federolf et al. (2018), both, addressed the topic in a special issue and provided their view. Both publications are part of the reviewed manuscript.

2. Purpose: Same comment from objectives. The purpose of your study is to investigate whether running kinematics and muscle activity are significantly different between shoes. Your purpose statement as written is unscientific and wordy.

The purpose statement has been revised.

Methods

1. Do you know the participants average running volume per week? A minimum of 2 runs per week is a very low standard. If you have the data, please provide the average running distance per week of your participants.

While the authors agree that this information would provide benefit, this data was, unfortunately, not collected. It is evident within the manuscript, however, that participation was open to a vide range of running experience. As a results, participants ranged from absolute beginners to elite runners.

2. From what I looked up, the Mizuno Wave Rider has an approximate weight of about 0.28 kg – assuming this is a typo, please correct. The Wave Universe, from the specs I looked up, is .08 kg. Please confirm the mass you stated is correct. Stating the source of your specs would be appreciated as well.

The authors thank the reviewer for point out the mistake. The footwear specifications provided within this manuscript have been revised and are in line with the previous publication by Nigg and colleagues (2017) that used the same footwear and has been cited multiple times throughout the manuscript.

3. The picture quality of Figure 2 is a little blurry. If possible, please update.

The picture the reviewers refer to has been removed in the revised version as no supporting information was provided by it.

4. What do you mean that you removed potential outliers? More detail is needed here. What data were you looking at for outliers? How was an outlier defined?

The revised manuscript provides exemplary kinematic trajectories for the ankle and knee joint, highlighting the quality of data that was included in the analysis. Further, the statement regarding the removal of outliers has been revised.

5. The kinematic comparison is not clear. First, I would argue that I should not need to refer to your previous study to see your methodology. Second, what do you mean by “mean absolute differences in 3D joint angles”? Did you interpolate your data to 100% of stance phase and then average all 101 data points to find the average joint angle during stance phase? If so, please state this in your description. If not, your methodology is unclear and needs to be expanded. I did read your previous study, but was still not clear on your technique.

The authors thank the reviewer for highlighting that the description was not clear. The statement in question has since been revised. However, the authors disagree with the reviewer on the topic of citing previous work that highlights the methodological approach. First, the novelty of this manuscript lies within the EMG analysis, which is described in detail. The kinematic analysis, while a necessary step to obtain the results, is identical in it’s approach and outcomes to the previous work that was cited extensively. Second, it is common practice in the scientific community to point readers to the respective works that provide greater details on specific analysis steps while stating only a brief summary. This approach shortens the presented work and interested readers know where to find greater details. Finally, the applied kinematic analysis is standard within the field of biomechanics. As the reviewer was able to infer correctly from the unrevised statement, movement trajectories were interpolated to 100 % of stance phase and averaged after calculating the difference. These reasons provide, in the opinion of the authors, sufficient justification to cite previous work highlighting specifics in respective analysis steps.

6. Can you cite or elaborate why 2 degrees was selected as your threshold?

The argument for the 2° threshold is that differences in joint movement below this threshold fall below the degree of reliability for skin marker-based 3D motion capture. Further, the work by Nigg and colleagues also highlights that the results for the 3° and 5° thresholds are essentially identical, independent of the threshold chosen. However, the revised manuscript selected a larger threshold (3º) to reflect clinical relevance. the somewhat arbitrary choice of a 3º threshold is scrutinized within the discussion section of the revised manuscript.

7. What is a whitening process?

A whitening process describes a technique that rescales a set of datapoints to an even spread. For example, dividing a dataset by its standard deviation describes a whitening process. This is, however, not applicable to the revised manuscript as the applied methodology has been updated and does not include this process anymore.

8. I do not understand the purpose of the PCA / SVM classifier. If you want to see differences between shoes for kinematics and EMG, why did you not simply use an ANOVA and look at significant differences between conditions? A justification for these advanced techniques is needed, specifically stating why simpler statistical methods would be insufficient (I would argue that simpler techniques are plenty sufficient).

The authors agree with the reviewer’s assessment and have since updated the methodological approach for this work.

Results

1. See major comment

As suggested by the authors the entire section has been rewritten.

Discussion

1. I’m withholding any specific comments here as I believe this section would need to be re-written if the authors address my previous comments and concerns

As suggested by the authors the entire section has been rewritten.
