# Supplementary material for: Influence of running shoes on muscle activity
Source: PLoS One. 2020 Oct 7;15(10):e0239852. doi: 10.1371/journal.pone.0239852 (PMC7540877; doi:10.1371/journal.pone.0239852)
Supplement: Attachment — Submitted filename: 2020-05 - PMP and EMG - R1 - Response to the reviewers.docx [file pone.0239852.s003.docx]

**FOREWORD**

First and foremost, we (the authors) would like to thank the reviewers for their invested time and effort. We understand that reviewing a document is a demanding task and thus we greatly appreciate the reviewers’ comments. We addressed each comment carefully and provided a comment specific answer. In most case we updated the manuscript according to the reviewers’ suggestions and, as a result, we feel that the manuscript is now stronger. In the few cases where our opinion differed from the opinion of the reviewers, we provided an extended answer, aiming to highlight our point of view. We hope that the reviewers will find that all comments have been addressed adequately. We greatly appreciate the reviewers’ contributions that guided us through this revision process.

**EDITOR**

Please ensure that you refer to Figure 3 in your text as, if accepted, production will need this reference to link the reader to the figure.

Figure 3 is referred to as Fig 3 in line 185 in the manuscript.

**REVIWER 1**

Summary

The authors sought to differentiate those who would alter their kinematics when exposed to different running shod vs. barefoot, and then explore the impact of different shoes on muscle activation in those with altered (>3deg) vs. those minimally altered (<3deg). Those with larger alterations in kinematics experienced larger changes in muscle activation, versus those who better maintained kinematics had minimal changes with altered shoes, and perhaps lesser changes with shod vs. barefoot. The study is generally well designed and written with findings presented in an appropriate manner.

General

Perhaps a matter of style, but in this reviewers’ opinion the manuscript is slightly askew in style. Specifically, the abstract is completely devoid of data, not even a mention of a % difference in muscle activity between those with “large” or small deviations in kinematics with alteration in shod status. Second, the introduction seems about a page too long, and the discussion a tad short.

We updated the abstract to include data (Lines 31 – 35). Additionally, the introduction was shortened and the discussion was extended.

Further, the abstract is quite vague and hard discern what the authors actually did, specifically line 24 “when a novel movement path was chosen”. I would strongly encourage the authors to better clarify here and elsewhere. To me, you stratified based upon kinematic differences between shod and unshod running, and differences with running in different shoes exploring the corresponding muscle activation. Another example is line 31 “when running conditions were different” and “when running conditions were similar”. Be specific, please. In this line, running conditions refers to two different concepts: shod vs unshod, and differences in shoe. Grammatically there is no concern, content wise the information is unnecessarily obscured.

We updated the abstract to be more precise. Specifically, results where included (e.g., Line 32) and statements were revised to be more precise (e.g., Line 31).

There is no obvious rationale for the shoes chosen/compared. Even if practical in nature please provide the readers some rationale as to why these three shoes, the descriptions are somewhat helpful but would be bolstered by additional information (e.g. exact models). To those who don’t partake in the ‘mizuniverse’, some context would be helpful.

Following the reviewer’s advice, we provided further information on each of the selected footwear models (Table 1). We also included a rational as to why these specific models were selected for this study (lines 121 - 122). Specifically, these models represent a range of footwear solutions available to the recreation runner. This was important since the purpose of this study was to assess the applicability of the preferred movement path paradigm to general and available footwear solutions.

It seems all conditions might be “altered” running condition, as they never ran in their “native shoe”. Please comment.

The reviewer is correct in assuming that all running conditions could be considered ‘altered’. We assume that the reviewers’ comment is directed at the concern that we potentially did not assess a runners ‘real’ preferred movement path as participants never ran in their ‘native’ shoe. This is an interesting train of thought. The preferred movement path pardigm suggests that a movement path is preferred only under a set of given constraints (i.e., running conditions, injury status, etc.). As a result, there is no single global ‘real’ preferred movement path. Instead the movement path that is most preferred is always dependent on the set of constraints. Various footwear constructions might be similar enough, and as a result, the movement path that is most preferred is the same one across these similar footwear constructions. It could also happen, however, that footwear constructions are not similar enough and, therefore, a different movement path would be preferred across these different footwear constructions. Considering this, we feel that it does not weaken the present study that no ‘native’ shoe was used. In fact, by providing all participants with the same footwear conditions we were able to systematically control the changes that a new set of footwear would introduce. In other words, we were able to control how similar / different two footwear conditions were. As a result, we were able to test the hypothesis that similar footwear constructions would result in the same preferred movement path but would induce different responses within muscle activation. We do agree with the reviewer and believe that it would be of great interest if future studies were to include a ‘native’ condition and observe the resulting interactions.

Were these shoes new and/or “broken in” before the first participant? Please clarify.

The footwear models were exclusively used for participants of this study. Shoes were available in multiple sizes, and for each size multiple pairs were available. As a result, participants ran in either a brand-new model or a model used for previous sessions of this study. However, no model was used for more than three participants. A clarifying statement has been added to the manuscript (Lines 125 – 127).

I assume all conditions were run in a single visit? This is unclear, please clarify.

Yes, data collection took place during a single visit within the laboratory. We added a clarifying statement to the manuscript (Line 117).

I fundamentally struggle with the term “preferred movement path”, it seems it IS their movement path, whether it is preferred or not might be a matter of debate. As a relatively equal number of men and women were recruited, an assumed differing Q angle of the women might be playing into differing kinematics and movement patterns which has been shown to relate to injury. Moreover, perhaps independent of sex, running kinematics likely plays a role in injury development (<https://link.springer.com/article/10.1007/s42452-019-0695-x> ), is their movement pattern preferred if it is predisposing them to an injury? I understand the sentiment, but preferred almost seems unnecessary, at least in this context or even misleading. Further in a number of instances the authors outrightly state athletes “choose” their PMP. Are the authors suggesting this is a conscious decision? Did you survey the athletes to ask them about their decisions? Kidding aside, their movement pattern might be also the result of a prior injury or surgery and not necessarily to avoid an injury (line 258), which could not be captured in the 6-month screening criteria. Individual movement patterns also likely depend upon sex/hormonal status, which is ignored in the current paper. The authors model proposes far more voluntary thought than is likely occurring, unless I under-think when I run.

We are unsure how to clearly address this comment. The first question the reviewer poses is whether a movement pattern is preferred if it is predisposing runners to an injury. The idea of a preferred movement path is that it resembles a running pattern that is preferred (i.e., the optimal solution to the problem of locomotion) under a given set of constraints. This optimization that the locomotor system attempts is not a trivial task. In a bad example it could happen, therefore, that the optimized solution might increase injury risks rather than reduce risks. For instance, considering a motivational aspect, if one needs to run fast to catch a leaving train the locomotor system would most certainly not optimize for a reduced risk of injury. It would rather optimize for increased speed, which might result in reduced stability, increasing the risk for injury. Under the certain constraints (motivation to catch the train) the resulting movement path would still be preferred, despite it not reducing the risk for injury in that instance. Further, we agree with the reviewer on the topic of previous injuries. Most certainly, previous injuries and / or surgeries would alter an individual’s preferred movement path. This aspect, however, is covered when one considers that the preferred movement path is the optimal solution to the problem of locomotion under a given set of constraints. In other words, a previous injury / surgery would define a specific constraint that the locomotor system accounts for when adopting a movement path that is preferred.

The second point raised by the reviewer concerns the phrasing that a runner ‘chooses’ their preferred movement path. We understand that this might be misleading. Certainly, we did not intent to say that it is a voluntary act to choose a preferred movement path. It is rather a subconscious process monitored by the locomotor system with very little (i.e., motivation) to no voluntary control by the runner. Nonetheless, the locomotor system chooses a movement path (the preferred one) out of all the possible solutions. We provided additional statements throughout the manuscript to clarify this interaction (e.g., Line 272).

The last point the reviewer touches on is the role hormones play in the scope of the preferred movement path. While the field of biomechanics rarely investigates hormonal interactions (this lies more within the regime of biochemistry) we cannot neglect that hormones play a role in our daily behavior. As such we would speculate that they do have an influence, as to what that influence is, however, lies outside of our expertise. Thus, we can unfortunately not provide much clarification here.

The paradigm suggests optimization to lower energy cost it is unfortunate to have missed the opportunity to quantify this in any form to substantiate these claims.

The paradigm suggests that optimization with respect to energy cost is one of many possible optimizations. Other possibilities include injury prevention, increased comfort, fatigue or maybe external factors such as motivation. The purpose of the current manuscript was not to prove (or disprove) a possible optimization towards energy consumption. It was more so to understand how a locomotor system is able to maintain a movement path. To this end, muscle activation patterns were investigated. We agree, however, that it would be an interesting investigation to focus on energy optimization. Thus, we added a statement proposing future research opportunities at the end of the discussion (Lines 356 – 360).

Line 169-170 the authors should provide rationale for this cutoff, perhaps citing the previous work of one of the authors. Presently it appears arbitrary, which is confirmed in the discussion (line 300).

We agree that a justification for this cut-off was missing. We since added a citation to the previous work of Nigg et al. (as suggested by the reviewer) and a statement highlighting this cut-off as a conservative threshold for clinical relevance (Lines 185 – 188).

The statistical approach is somewhat confusing. If the authors are seeking to compare the groups, then shouldn’t this factor be included in a singular model rather signed rank tests “separate for each group”, such as a two-way ANOVA, to gain insight into potential interactions of group and running task? Based upon this the authors haven’t factually compared the groups and should refrain from such comparisons.

The focus of this work was to investigate changes in muscle activation in runners who showed small / large kinematic differences in six footwear comparisons. In other words, interactions between runners who showed small kinematic differences and those who showed large kinematic differences were not the immediate focus. We were simply interested whether runners who showed small / large kinematic differences in any given comparison of two footwear conditions would show small or large changes in EMG activity. Based on this, we believe that applying an ANOVA would be a mistake. We understand, however, that the previous phrasing of the purpose of the study and the phrasing of the statistical approach might have been misleading. To clarify our goal and therefore strengthen the choice of the statistical test, we rephrased the purpose of the study (Lines 96 – 102) and the statistics section of the study (Lines 216 – 220).

How is an absolute change a % when based upon change in EMG AUC? Include the calculation or perhaps the author mean “relative change”? Further, the data analysis section doesn’t seem to match the results section

We clarified this statement. It now expresses that we calculated mean absolute differences and expressed those with respect the first of the two running conditions that were compared (Lines 208 – 213).

We further updated the entire method section to clearly reflect what is presented in the results section.

Minor

Line 253 “patter” to pattern

This change has been addressed (lines 274).

Line 310 “constrains” constraints

This change has been addressed (lines 307).

In the acknowledgements it seems there is a remnant from an MSSE submission, please remove.

This change has been addressed (lines 372).

Recent work in women suggests an increase in EMG with barefoot running as compared to shod (PMID 31839842), and seems a relevant reference to include.

We agree with the reviewer and have added the suggested citation (lines 89).

**REVIEWER 2**

General Comments

This study was on whether muscle activity alterations can be a reflection of changes in movement path or running shoe conditions. This study was designed to test a new paradigm of running mechanics that explains that the cause of injury may not necessarily be directly associated with impact forces or increased pronation but could be the result of elevated changes in internal forces. Overall, the structure and style of this manuscript was very good. It was easy to follow and presented a new, interesting idea on running biomechanics. I had very few concerns about this paper, which are discussed below.

Specific Comments

METHODS

Line 119: Was there rationale for only including heel-to-toe runners in this study? It was discussed later on that you wanted to have a study that consisted of a heterogeneous population, so I was wondering why forefoot or midfoot runners were not also included. I know the study was looking at EMG changes right before and after heel-strike, but I was thinking this paradigm could hopefully be tested and applied to all types of runners (i.e. striking patterns and running ability).

We agree with the reviewer’s assessment that this paradigm should be applicable to all running styles (rearfoot, midfoot, and forefoot strikers). Unfortunately, comparing foot strike patterns (e.g., rearfoot vs. forefoot) from a biomechanical perspective shows that these are drastically different from one another. Based on this, a much larger sample size would have been necessary to derive meaningful conclusions for each individual strike pattern. By focusing on a single running style (rearfoot strikers), we were able to finish this study in a reasonable amount of time (smaller sample size). Additionally, rearfoot striking is the most common pattern amongst recreational runners and, therefore, presents the best opportunity for new insights.

A clarifying statement has been added to the manuscript (Lines 107 – 109).

DISCUSSION

Line 244: You discussed that the preferred movement path paradigm has been proposed as a replacement of the traditional paradigms, but starting at line 54 in the introduction, you stated that this new paradigm has been introduced to further enhance traditional understanding. I was just confused by this connection because of the wording. Is this novel paradigm designed to replace or strengthen the previous paradigms?

We agree with the reviewer’s assessment that the wording is confusing. The paradigm was introduced to replace the old paradigms (pronation and impact force). To this end it aims to provide a novel perspective on running biomechanics that is based on a more functional understanding of running. We updated the statements mentioned by the reviewer to better reflect this connection (Lines 54 – 55 and 266 – 267).

Line 253: change ‘patter’ to ‘pattern’

This change has been addressed (lines 274).

Line 256: What was the purpose of using ‘most economical’ when describing the reasoning for why a preferred path is important? Does ‘economical’ refer to max VO2 or energy consumption? If it does not refer to just energy efficiencies, what does it entail? The reason I ask is because a few sentences following, you discussed that one of the reasons why a preferred movement path is selected is to reduce energy consumption, while in Line 256 you state that it is not directly correlated with running economy. I may be misinterpreting the statement, but I associate a higher running economy with lower levels of energy consumption.

We see how the wording might have caused confusion. Within this paragraph we aimed to state that the locomotory system optimizes for multiple factors instead of just one. There has been a prevalent notion in the field of running biomechanics that suggests that runners constantly optimize for reduced energy consumption. The paradigm of the preferred movement path, however, aims to broaden this perspective by suggesting that the optimization purpose of the locomotor system is influenced by multiple factors and not just reduced energy consumption. Possible optimization criteria might include an increased feeling of comfort, a reduction in perceived pain, and / or a reduction in the potential risk of injury in addition to a reduction in energy consumption. We attempted to clarify this interaction by rewording the statement (Lines 278 – 282).

**REVIEWER #3:**

General Comments

This manuscript attempts to shed light on the preferred movement path, a novel idea which may help with understanding relationships between running shoe design, running mechanics, and injury. Given the topic, this would be of interest to readers and important for the field. That said, I have some comments and concerns before this manuscript could be accepted for publication.

This is a revised version. While it is much improved over the previous versions, the authors have not utilized the previous reviewers comments fully to improve the manuscript. Rather, there are superficial acknowledgements of the reviewer comments and superficial fixes instead of meaningful discussion. More specifically, the authors have not adequately addressed the following:

• Reviewer 1’s question regarding how the use of the preferred movement path could inform shoe design and/or reduce incidence of running injuries.

We believe that footwear constructions that naturally support a runners preferred movement path may reduce the risk for injury as it potentially reduces load on less adapted internal structures. This assumption is in line with recent findings by Willwacher and Colleagues (DOI: 10.1038/s41598-020-58352-5) who showed that footwear promoting an individual’s habitual motion pattern resulted in reduced cartilage loss after a prolonged run. We provided a citation and explanatory statement within the manuscript to clarify this interaction (Lines 61 – 63).

• Both reviewer 1 and 2 requested more details regarding how outliers were handled and criteria for removing data. This has not been provided.

We believe that the use of the term ‘outliers’ might have led to confusion. When we reported that all trials were visually inspected to detect and remove outliers, we intended to highlight that running trials that did not represent a clear rearfoot strike pattern were removed. Similarly, EMG data that displayed clear movement artifacts (i.e., low frequency components of high intensity) were removed as well. We most certainly did not intend to say that participants who showed different behaviors in EMG or kinematics were removed. In other words, we cleaned the data by removing trials that displayed noisy and / or unusable data.

We provided an additional statement to highlight these steps (Lines 166 – 169).

• Reviewer 1’s request for the authors to comment on whether similar results would occur following an adaptation period.

A paragraph highlighting possible implications of an adaptation period has been added (Lines 341 – 351).

• Reviewer’s 2 suggestion to focus on the shod vs barefoot instead of the shod vs shod comparisons. The authors answer this saying this has already been done, but it has not since it is not possible to actually determine which movement path is the preferred.

Here the current reviewer refers to a comment made with respect to the initial submission of this manuscript. The comment (made by one of the previous reviewers) was based on the assumption that a runners’ preferred movement path is the movement pattern exhibited during barefoot running. Following this logic, the previous reviewer suggested that a comparison between barefoot and shod running should be the focus of this manuscript. We, however, believe that this logic is flawed. A runners’ preferred movement path is not exclusively the movement pattern exhibited during barefoot running. In fact, we believe that a runner may change their preferred movement depending on factors such as the current training status, the current footwear condition, the current presence / absence of injury, and / or the current fatigue level. This belief is clearly expressed in the introduction of the current version of the manuscript (Lines 66 – 69). In fact, a previous study by Nigg and colleagues (DOI: 10.1249/MSS.0000000000001260) found that the majority of runners maintained their preferred movement path when switching between similar footwear constructions but adopted a different preferred movement path when changing from shod to barefoot running. It remained unclear, however, how runners were able / unable to maintain a preferred movement path, and whether certain shoe constructions benefitted a preferred movement path. We aimed to address these questions with the current manuscript by investigating changes in muscle activation across barefoot running and running in different footwear constructions. This approach allowed us to speculate about the role of muscle activation when a preferred movement path was different in two conditions (i.e., barefoot and shod), and when a preferred movement path was the same in two conditions (i.e., shod and shod).

If we had focused exclusively on barefoot to shod comparisons (as suggested by the reviewer) we would not have been able to speculate about the effects of similar shoe constructions on a runner’s preferred movement path. We therefore believe that our approach provides more insights than the approach suggested by the previous reviewer.

• Reviewer 2’s comment about the EMG data analysis. The authors have not simplified the analysis or presented it in a way most readers would understand. They certainly did not do the analysis as reviewer 2 suggested. If they are not then at a minimum in the methods they need to justify why their approach is more insightful or a more appropriate approach.

We applied a wavelet transform to the raw EMG signals to investigate the signals within a time-frequency space. This process in well established in EMG analysis (e.g., DOI: 10.1109/TNSRE.2003.819901, or DOI: 10.2478/v10048-011-0009-y) and provides valuable insights to EMG analyses that traditional methods (e.g., amplitude, or envelope) cannot provide. Further, in this specific case it allowed us to identify movement artifacts within EMG signals and reduce their effect on the outcome variable (area under the curve), contributing to a clean data set.

Investigating the area under the curve, as opposed to the amplitude (which is time dependent) or simply the onset / offset of EMG, results in a better estimate of overall EMG activity. We believe that the area under the curve is, therefore, the best approach for the purpose of this manuscript.

The analysis in itself is a straightforward process described in detail in the cited publications. Additionally, it is well established in the field of biomechanics and we provided the reader with all necessary information to replicate the approach. We did rephrase the paragraph aiming for a more concise explanation (Lines 201 – 213).

• Reviewer 2’s comment about the results section being insufficient. The authors have superficially addresses this but still do not provide mean values or measures of effect size.

The manuscript clearly states mean values for changes in muscle activation throughout the results section. These can be found in Lines 236, 248, and 251. For example, it states that across barefoot to shod comparisons (i.e., Barefoot vs. Rider, Barefoot vs. Be, Barefoot vs. Universe) mean changes in the activity of the TA, PL, GM, SO, VL, and BF were in the order of magnitude of 35%, 11%, 17%, 10%, 27%, and 16%, respectively, for runners who showed kinematic changes of less than / equal to 3 (Lines 235 – 237).

Reporting effect sizes, however, is a more difficult topic. We understand that effect sizes are a valuable contribution to p-values as their purpose is to provide the reader with an idea about the magnitude of change that is not included in a simple report of a p-value. Further, in a parametric scenario (t-test / ANOVA) the interpretation of an effect size (e.g., cohen’s d) is straight forward and provides additional information. In a non-parametric scenario, however, one cannot simply report the same effect sizes, as they are adversely affected by departures from normality and heterogeneity of variances. Put simply, reporting effect sizes may not be well advised for non-parametric data sets such as the one in this manuscript. Considering, however, that the reported values in this manuscript are averaged changes in percentages the reader is, in our opinion, provided with an estimate about the magnitude of the effect. Additionally, the provided graphs (Fig 4 and Fig 5) give visual comparison for the changes observed.

Given that effect sizes for not parametric data sets are not well established, and that the reported mean values in combination with the reported figures provide the reader with a good overview on the magnitude of changes, we are of the opinion that the reader is provided with similar information that a parametric based effect size calculation (e.g., Cohen’s d) would provide.

• Reviewer 2’s concern about potential bias given that the authors are the only group who has investigated or suggested the preferred movement path. This requires substantial attention and should be discussed openly in the limitations of this manuscript.

We added a discussion on the implications of a potential research bias as suggested by the reviewer (Lines 351 – 356).

• Reviewer 2’s comment regarding more details being needed regarding the kinematic analysis. I am in agreement with reviewer 2 that it is not sufficient to simply cite the previous paper. This should stand on its own as an independent work. Additional details should be provided.

In addition to the citation of the previous work, we provided more details on the analysis of kinematic data and how this data were obtained (Lines 139 – 150 and 174 – 188, respectively). In our opinion, the work can now stand on its own (as suggested by the reviewer) while still providing an indication to where this approach was initially published.

In addition to the above comments, I have several general comments that were not raised previously which I believe need to be addressed before this can be published.

• Related to reviewer 2’s concern about potential scientific bias, there is another group which is working on a hypothesis similar to the PMP. While the authors have cited Trudeau and colleagues first paper, they have done so in a very superficial manner and not incorporated the main points of Trudeau et al.’s work. This is an important element to discuss as, in contrast to the PMP, Trudeau and colleagues suggest a method for quantifying the habitual movement path and for assessing how much a shoe promotes or changes this path. They have also recently published a paper showing that a long run in shoes which do not promote the habitual movement path results in changes to cartilage volume as measured by MRI. At a minimum this new paper should be included in the introduction, most likely around the discussion in lines 62-64.

As suggested by the reviewer, we included Trudeaus work into the manuscript (Lines 61 – 63 and 329 – 331).

• The reviewers discuss the use of changes greater or less than 3 degrees as a cutoff for deciding whether individuals changed their movement path. Why have the authors not used some form of entire curve fitting comparison? It would seem that by using a somewhat arbitrary magnitude value the authors could end up with a participant in the substantially changes group where really the only thing that changed is the magnitude of the movement. Alternatively, the entire curve could still be shifted. However, the curve itself may have a very similar overall shape and trajectory. Indeed, this appears to be the case in most of the example curves presented in Figure 2, which should show the different movement paths. Really the ankle frontal plane motion is the only curve which looks different across conditions. All the other curves are simply offset.

We agree with the reviewer that different approaches to determine changes in kinematics proves a valuable research for future studies. Thus, the revised discussion section does encourage future research to explore other methods of quantifying differences (Lines 335 – 339). In this study specifically, we aimed to follow the example put forward by Nigg and colleagues, as this study was intended as a follow-up to the original one.

• Did all runners maintain a consistent foot strike pattern in the barefoot and shod conditions? If not one could reasonably expect different kinematics as this has been shown many times. But is that really showing a difference in preferred pattern, or simply a difference between footwear conditions?

Yes, all runners maintained a consistent rearfoot strike patter. When a trial did not contain a clear rearfoot strike pattern, it was repeated and not included in the final data set. Overall, participants were required to repeat trials until 10 valid rearfoot strikes were collected. A clarifying statement was added to the manuscript (lines 127 – 130).

• How do the participants in Table 1 match with the data presented in Figures 4 and 5? It is not clear which participants from which groups are shown in Figures 4 and 5.

We provided sample sizes for the individual comparisons in Figures 4 and 5. These match with the sample sizes represented in Table 2 and give a clear indication which participants are represented in each graph.

Specific Comments:

Abstract, lines 30 – 37. These are wordy and difficult to follow as currently written. Suggest revising for clarity.

The abstract has been revised and reworded (Lines 22 – 37).

Line 42 – What do the authors consider “excessive” impact forces or pronation. While their point is well made that these are the dominant paradigms, is the problem really with the paradigms or with the use of the term “excessive” amount where there is no agreement on what constitutes excessive? Perhaps higher levels would be a better phrase?

We agree that the term excessive leaves room for interpretation. Aiming to be more precise with this statement, the phrasing was changed to ‘higher magnitudes’ (lines 42). We believe that the updated phrasing clearly highlights that impact forces and / or pronation of higher magnitudes were speculated to be harmful to the human body. The reverse of this phrasing highlights that lower magnitudes might not be harmful, in fact it could be argued that these are natural as they are inherent to running.

Line 59 – Here the authors suggest runners maintain a given movement path when changing between different shoe conditions. Yet, in line 67 the authors suggest that the movement pattern might be different in running shoes verse ski boots. These appear to be presenting opposite views.

We can see why this phrasing might have caused confusion. The rational we aimed to express is that a preferred movement path remains constant in similar footwear constructions (e.g., various running shoes like cushioned or motion control). This reasoning would explain why many studies failed to find significant kinematic differences across many running shoes. When footwear constructions are substantially different (for instance a running shoe compared to a ski boot), however, each construction has its own preferred movement path. Running in a ski boot would most certainly show altered kinematic gait patterns when compared to running in a normal cushioned shoe. We aimed to clarify this interaction by rewording both statements within the manuscript (Lines 58 and 68).

Line 111 – What kinematic differences are the authors referring to? The entire curve? Peak values, range of motion? Some more detail is needed.

We updated this sentence to reflect clearly what kinematic differences were investigated. Specifically, it now reads ‘averaged absolute differences in the ankle and knee joint angle trajectories’ in line 98.

Lines 122 – 123 – The authors have opened the study to a wide cross section of runners. While this improves the external validity, the authors should also report the experience level of their participants. How much mileage were they running each week? What was average training paces? This would be especially important information to present in light of the discussion in lines 291 – 299 about experience level.

Unfortunately, we did not specifically collect the experience level and the weekly mileage of each runner. We do agree, however, that this information is of great interest. Reviewing the notes collected during testing we aimed to provide an estimate about the average distance for any given run (Lines 110 – 111). In combination with the minimum number of weekly runs that we reported (Line 109) this provides an estimate about the skill level of our participants. We further raised this point in the discussion (Lines 324 – 326).

Acknowledgements – There appears to be verbiage in here from a previous submission to an ACSM journal. If this is not required for PLoS One then it should be removed.

This change has been addressed (lines 372).
