# Supplementary material for: Influence of running shoes on muscle activity
Source: PLoS One. 2020 Oct 7;15(10):e0239852. doi: 10.1371/journal.pone.0239852 (PMC7540877; doi:10.1371/journal.pone.0239852)
Supplement: Attachment — Submitted filename: 2020-08 - PMP and EMG - R2 - Response to the reviewers.docx [file pone.0239852.s004.docx]

**REVIWER 1**

The authors have made significant improvements to the manuscript. However, a minor issue remains. Namely, in line 367 " ...in [ previous work?]. This needs an edit.

Thank you for your assessment. We greatly appreciate that our efforts to improve this manuscript have been well received. Following the reviewer’s suggestion, we have updated the respective line. The clean version of the manuscript now reads: ‘This threshold was selected as it represents a conservative threshold for clinical relevance and was suggested in previous work [10]’ (Line 291).

For your convenience, we highlighted this change in the version with tracked changes.
